# Novel anticoagulation therapy using apple watch after catheter ablation for atrial fibrillation—Up to AF trial: Design and rationale

**DOI:** 10.1002/joa3.13194

**Published:** 2024-12-05

**Authors:** Akihiro Sunaga, Nobuaki Tanaka, Yasuyuki Egami, Hitoshi Minamiguchi, Takafumi Oka, Masato Kawasaki, Koichi Inoue, Masaharu Masuda, Miwa Miyoshi, Nobuhiko Makino, Tetsuya Watanabe, Daisaku Nakatani, Katsuki Okada, Hirota Kida, Yuki Matsuoka, Daisuke Sakamoto, Tetsuhisa Kitamura, Tomomi Yamada, Yohei Sotomi, Yasushi Sakata, the OCVC‐Arrhythmia Investigators

**Affiliations:** ^1^ Department of Cardiovascular Medicine Osaka University Graduate School of Medicine Osaka Japan; ^2^ Cardiovascular Center Sakurabashi Watanabe Hospital Osaka Japan; ^3^ Division of Cardiology Osaka Rosai Hospital Sakai Japan; ^4^ Cardiovascular Division Osaka Police Hospital Osaka Japan; ^5^ Division of Cardiology Osaka General Medical Center Osaka Japan; ^6^ Cardiovascular Division National Hospital Organization Osaka National Hospital Osaka Japan; ^7^ Cardiovascular Center Kansai Rosai Hospital Amagasaki Japan; ^8^ Department of Cardiology Osaka Hospital, Japan Community Healthcare Organization Osaka Japan; ^9^ Department of Cardiology Rinku General Medical Center Izumisano Japan; ^10^ Department of Medical Informatics Osaka University Graduate School of Medicine Osaka Japan; ^11^ Department of Social and Environmental Medicine Osaka University Graduate School of Medicine Osaka Japan; ^12^ Department of Medical Innovation Osaka University Hospital Suita Japan

**Keywords:** Apple Watch, atrial fibrillation, DOAC, tailored anticoagulation

## Abstract

**Background:**

Continuous anticoagulation based on the CHA2DS2‐VASc score is recommended to prevent embolism caused by atrial fibrillation (AF), but it does not consider AF episodes. The Apple Watch's continuous heart rhythm monitoring and fast‐acting direct oral anticoagulants (DOACs) could enable precise, episode‐tailored anticoagulation, reducing bleeding risks while preventing stroke. This study evaluates Apple Watch‐guided personalized anticoagulation therapy, adjusting DOAC usage based on real‐time AF detection.

**Methods:**

This multicenter prospective single‐arm study will enroll patients who have maintained sinus rhythm post‐ablation and are on DOACs. The target enrollment is 50 patients free of AF for at least 30 days following the initiation of Apple Watch monitoring. If no AF occurs for the first 30 days of monitoring, anticoagulants will be discontinued on day 31. If AF is confirmed after day 31, DOAC administration will be resumed and continued until the end of the observation period. The primary endpoint is the reduction in the total number of days with DOACs from day 31 to day 360 compared to the conventional method of continuing anticoagulation. Secondary endpoints include all‐cause mortality, stroke, systemic thromboembolism, bleeding events, and Apple Watch malfunctions.

**Results:**

Enrollment of a total of 50 patients was completed in April 2024. Follow‐up of the last enrolled patient will be completed in April 2025 and primary results are expected to be available in late 2025.

**Conclusions:**

The Up to AF trial is the first trial to evaluate Apple Watch‐guided personalized anticoagulation therapy. This trial represents a potential advancement in personalized medicine for AF management.

## Graphical abstract

The Up to AF trial evaluates Apple Watch‐guided personalized anticoagulation therapy for atrial fibrillation (AF), adjusting DOAC use based on real‐time AF detection. In this single‐arm study, 50 post‐ablation patients are monitored, aiming to reduce DOAC days while assessing mortality, stroke, and bleeding events. Results are expected in 2025.

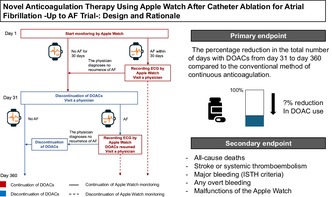

## 1 INTRODUCTION

More than 33 million individuals globally are afflicted by atrial fibrillation (AF) [1], with an estimated incidence of exceeding 10% in those over the age of 70 [2]. Numerous complications are associated with AF, including stroke, which can significantly impact prognosis. The effective management of AF is a pressing issue in an aging society. To prevent embolism caused by AF, anticoagulant therapy is performed based on the CHA_2_DS_2_‐VASc score, which is a risk assessment tool for cerebrovascular events in AF patients [3]. The long‐term continuation of post‐ablation anticoagulation therapy is recommended to be guided by the patient's stroke risk profile, without considering AF burden [4]. This approach is necessitated by the often brief and asymptomatic nature of AF episodes, making timely recognition and intervention challenging.

Advances in devices capable of continuous heartbeat monitoring have demonstrated that embolic risk varies with AF burden [5]. As continuous anticoagulation increases bleeding events [6], it may be detrimental with minimal benefit for those with no or minimal AF burden. Traditionally, anticoagulant therapy involves the use of warfarin, which requires weeks to achieve therapeutic anticoagulant effects. However, the recent introduction of direct oral anticoagulants (DOACs) allows for the rapid attainment of anticoagulant effects within hours of oral administration. Consequently, while warfarin must be administered even in the absence of AF to maintain therapeutic blood concentrations for when AF occurs, DOACs can be administered upon the onset of AF.

Studies utilizing implantable cardiac monitors or devices to determine the administration or discontinuation of DOACs based on the presence or absence of AF have demonstrated a reduction in DOAC use by 74.6% to 94% [7, 8], suggesting that current practices unnecessarily increase bleeding risk and healthcare costs. Implantable cardiac monitors and devices are invasive, and only the remote monitoring provider has information about AF onset, causing delays in DOAC administration. Recently, non‐invasive devices capable of continuous monitoring, heart rate analysis, and ECG functions have been developed. These devices alert users to abnormal heartbeats in real time, allowing for immediate action. We have chosen to use one of these devices, the Apple Watch, to investigate the efficacy and safety of Apple Watch‐guided personalized anticoagulation therapy.

## 2 METHODS

### 2.1 Objective

In patients who maintain sinus rhythm following ablation for AF, we will investigate whether Apple Watch‐guided personalized anticoagulation therapy—where DOACs are continued or discontinued based on the presence or absence of AF as monitored by the Apple Watch—will reduce DOAC usage compared to the conventional method of continuous anticoagulation.

### 2.2 Study design

**FIGURE 1:**
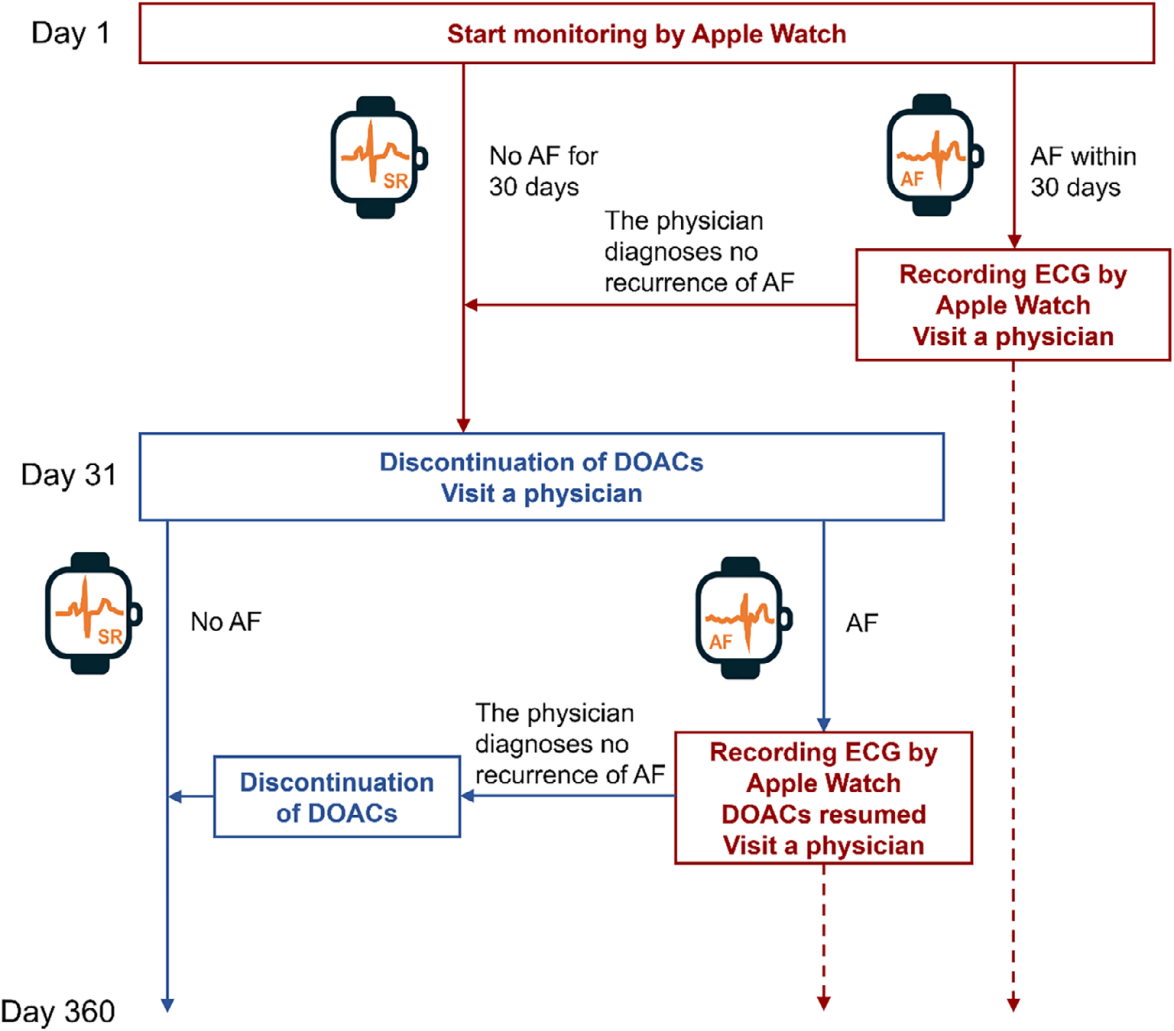
Study design. The red line indicates continuation of DOACs, the blue line indicates discontinuation of DOACs, the solid line indicates continuation of monitoring by the Apple Watch, and the dashed line indicates the discontinuation of monitoring by the Apple Watch. Heartbeat notification is defined as high heart rate notification and irregular heartbeat notification. AF, atrial fibrillation; DOAC, direct oral anticoagulant; ECG, electrocardiogram.

The study design is a multicenter, prospective, single‐arm intervention study. After confirming that the study patients meet the inclusion and exclusion criteria, consent will be obtained, and cases will be enrolled via the case registration system. After enrollment, the patients will wear an Apple Watch and begin monitoring for AF **(**Figure 1 **)**.

If no AF occurs for the first 30 days of monitoring, anticoagulants will be discontinued on day 31. AF is defined as the notification of irregular heartbeats and a high heart rate indicated by the Apple Watch, and AF, atrial flutter, and atrial tachycardia indicated by 12‐lead ECG or Holter ECG.

If AF is detected on the Apple Watch by day 30, the patient will obtain an ECG with the Apple Watch. Monitoring with the Apple Watch will then be discontinued. Thereafter the patient will see a physician within 7 days, and the physician will evaluate the ECG obtained by the patient with the Apple Watch. If the physician determines that the patient is not in AF, monitoring with the Apple Watch will be resumed. If the physician confirms that the patient is in AF, DOAC administration will be continued until the end of observation period.

If AF is detected on the Apple Watch after day 31, the patient will obtain an ECG with the Apple Watch. Monitoring by the Apple Watch will then be discontinued. At the same time, DOAC administration will be resumed. Thereafter, the patient will see a physician within 7 days, and the physician will evaluate the ECG obtained by the patient with the Apple Watch. If the physician determines that the patient is not in AF, DOAC administration will be discontinued and monitoring by the Apple Watch will be resumed. If the physician confirms that the patient is in AF, DOAC administration will be continued until the end of observation period.

If AF is confirmed by various ECG tests without notification from the Apple Watch, such as voluntary ECG recording by the ECG application on the Apple Watch or ECG tests at hospitals, the subject will be examined by a physician and, if determined to be in AF, will continue or resume taking DOACs and terminate the monitoring via the Apple Watch.

This study has been registered at the Japan Registry of Clinical Trials (jRCTs052230070) and adheres to the principles of the Declaration of Helsinki. The study protocol and informed consent forms have been reviewed and approved as a specific clinical trial in accordance with Clinical Trials Act by a certified review board (CRB), the Osaka University Clinical Research Review Committee (CRB5180007). All patients must provide informed written consent prior to participation in this study.

### 2.3 Apple Watch setting

Apple Watches from series 4 or later were used in this study. The Apple Watch can record heart rate using photoplethysmography and notify users when the heart rate is high, low, or irregular. High heart rate notification will be set to 120 bpm, and low heart rate notification will be disabled. The irregular rhythm notification will be enabled. The irregular rhythm notification can diagnose AF with a sensitivity of 78.9% and any arrhythmia, including AF, with a sensitivity of 98.2% [9]. ECG application implemented on the Apple Watch can diagnose AF with a sensitivity of 98.5% and specificity of 99.3% [9]. Both the irregular rhythm notification and ECG application are approved as medical devices by the Pharmaceuticals and Medical Devices Agency in Japan.

The Apple Watch needs to be recharged, and it will be recommended to allocate bath time for charging. It will be recommended to wear the watch even when sleeping, except during charging time.

### 2.4 Eligibility criteria

The study population will consist of patients who maintain sinus rhythm after ablation for AF and are taking DOACs. Inclusion and exclusion criteria are summarized in Table 1. A signed informed consent form approved by CRB will be obtained from each patient prior to any intervention.

**TABLE 1:** Eligibility criteria.

**Inclusion criteria**
No AF recurrence after 3 months following catheter ablation
Patients using DOACs
Patients using an iPhone (version 8 or later, SE second generation or later)
CHA_2_DS_2_‐VASc score of 3 or less
Age 22 years or older
Patients who can provide written consent
**Exclusion criteria**
Mechanical valve
Severe mitral stenosis
Cases in which anticoagulation is essential (deep venous thrombosis, intracardiac thrombus, etc.)
Resting heart rate of 100 beats/min or higher
Pacemaker implantation
Patients with a history of fatal arrhythmia (ventricular tachycardia, ventricular fibrillation) or with an implantable cardioverter defibrillator (ICD)
Post‐operative heart transplantation
Chronic hemodialysis
Patients with a history of cerebral infarction
Patients scheduled for elective surgery during the observation period, including catheterization
Patients who have difficulty operating the device due to visual impairment, cognitive impairment, etc.
Other subjects deemed inappropriate by the investigators

### 2.5 Data collection and follow‐up

At the time of informed consent, baseline data, medical history, comorbidities, medications, blood test, 12‐lead ECG, transthoracic echocardiogram, date of catheter ablation for AF, and type of AF prior to ablation will be collected. After the enrollment, patients will continue to wear the Apple Watch until the end of the observation period, 12 months after enrollment. Patients will be strongly encouraged to wear the Apple Watch even while sleeping.

Patients will be followed at 1, 3, 6, 9, and 12 months after enrolment. A 12‐lead ECG, alerts, and ECG of the Apple Watch, number of days wearing the Apple Watch, and DOAC medication status will be obtained at each follow‐up visit. Medication assessment will be performed at 12 months. Symptom‐driven 12‐lead ECG, Holter ECG, and any other devices capable of obtaining ECGs can be used at any time at the physician's discretion. Outcomes, adverse events, and malfunctions of the Apple Watch will be collected whenever they occur during the observation period.

### 2.6 Study endpoints

The primary endpoint of this study will be the percentage reduction in the total number of days with DOACs from day 31 to day 360 compared to the conventional method of continuous anticoagulation. Analysis of the primary endpoint will be calculated as follows: the denominator will be the observation period (person‐days) and the numerator will be the total number of days with DOACs during the observation period. The degree of decrease will be evaluated by calculating “1 ‐ the rate of DOAC use during the observation period”. The observation period used in this calculation will be from day 31 to day 360. For DOACs taken twice a day, if taken only in the morning or evening, the days with DOACs will be counted as 0.5 days.

The secondary endpoints will include all‐cause death, stroke, systemic thromboembolism, bleeding events, and malfunctions of the Apple Watch. Bleeding events will be defined as major bleeding according to the ISTH bleeding criteria [10] and any overt bleeding events other than the major bleeding. Stroke will be defined as a non‐traumatic focal neurologic deficit lasting more than 24 hours, diagnosed by brain imaging or autopsy as ischemic stroke [11]. Systemic thromboembolism will be defined as a clinical history consistent with acute loss of peripheral blood flow and findings of embolism through objective testing such as imaging studies and surgical specimens.

### 2.7 Research institutions: Osaka cardiovascular conference

The Osaka Cardiovascular Conference (OCVC) consists of the cardiologists affiliated with Osaka University Graduate School of Medicine and its associated hospitals. The OCVC was initiated in 2014 to address clinical questions in the realm of cardiovascular medicine. Among the 35 participating institutions, 9 hospitals that perform a large volume of invasive treatments for AF comprise the OCVC‐Arrhythmia investigators and will participate in this study.

### 2.8 Sample size setting

The target sample size is 50 patients who remain free of AF for at least 30 days following the initiation of monitoring with the Apple Watch. This was determined based on the available resources, including time, personnel, and budget limitations.

### 2.9 Statistical analysis

Patient background analysis will be performed on all patients, including those with AF within 30 days of the start of Apple watch monitoring. The primary and secondary endpoints will be analyzed in a population free of AF for at least 31 days after the start of monitoring by the Apple watch. All‐cause death, bleeding events, stroke, and systemic thromboembolism will be estimated by the Kaplan–Meier method. Those rates at 12 months and their 95% confidence intervals will be calculated using Greenwood's formula. Categorical variables were presented as counts (percentages) and compared using the chi‐squared test or Fisher's exact test, as appropriate. Continuous variables will be reported as mean (standard deviation) or median (interquartile range) and compared using the Student's t‐test, Mann–Whitney U test, or paired t‐test as appropriate. P‐values <0.05 will be considered statistically significant for superiority testing. Statistical analysis will be performed using R software (version 4.3.1 or higher; R Foundation for Statistical Computing).

### 2.10 Study status

**FIGURE 2:**
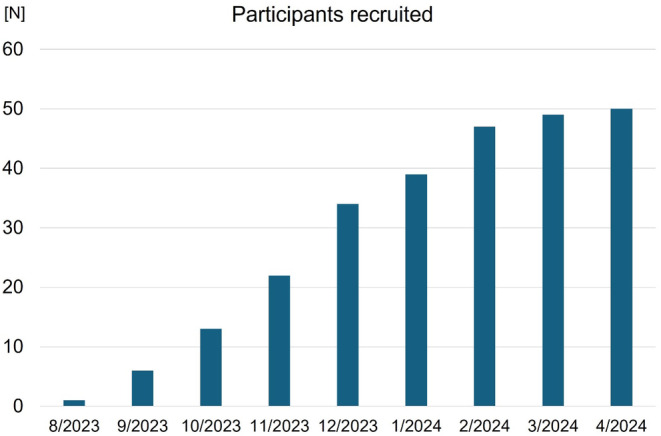
Timeline for patient enrollment in the Up to AF trial. The figure shows the cumulative number of participants enrolled in the Up to AF trial between August 2023 and April 2024.

Between July 2023 and April 2024, a total of 54 patients were enrolled from the 9 sites. The target sample size (*N* = 50) was achieved in April 2024 **(Figure** **2** **)**. Four patients experienced AF during the first 30 days, and remaining 50 patients are undergoing the Apple watch‐guided antithrombotic therapy. Follow‐up of the last enrolled patient will be completed in April 2025 and primary results are expected to be available in late 2025.

## 3 DISCUSSION

This Up to AF trial is designed to investigate whether Apple Watch‐guided personalized anticoagulation therapy—where DOACs are continued or discontinued depending on the presence or absence of AF monitored by the Apple Watch—will reduce the use of DOACs compared to the conventional method of continuous anticoagulation.

After catheter ablation for AF, the risk of bleeding and embolization with oral anticoagulation is 1.0 to 1.6% and 0.2 to 1.0% per year, respectively, while the risk of bleeding and embolization without oral anticoagulation is 0.02 to 0.8% and 0.03 to 0.6% per year, respectively [6, 12, 13]. Meta‐analyses have found that discontinuing anticoagulation reduces bleeding risk but does not change embolic risk [14, 15]. Of note, our study evaluates a safer and more effective treatment strategy than the previous blind therapy. Since no evidence of such strategy exists to date, and a large‐scale trial would be required to evaluate clinical outcomes of the strategy, we conducted the current pilot study with a surrogate of anticoagulant use.

The ASSERT study found that subclinical AF shorter than 24 hours in duration had no different embolic events than no subclinical AF, while subclinical AF longer than 24 hours in duration had significantly more embolic events compared to no subclinical AF [17]. The NOAH‐AFNET 6 study showed that in a population with a median atrial high‐rate episode duration of 2.8 hours, anticoagulation does not reduce embolic events and only promotes bleeding events [18]. Patients with low AF burden are thought to be at low risk of embolic events according to these studies. A study with continuous cardiac rhythm monitoring showed >98% AF burden reduction by catheter ablation [19]. Nevertheless, since there have been reports that embolic events were more frequent with CHA_2_DS_2_‐VASc score of 4 or higher [16], the selection criterion for this study was set to CHA_2_DS_2_‐VASc score 3 or lower for safety reasons.

## 4 LIMITATION

First, the Apple Watch will not provide notification during exercise. Second, it is not possible to monitor heart rate continuously 24 hours a day with the Apple Watch due to the need to remove it for recharging. Thirdly, although we can track the number of days the Apple Watch is worn, we cannot determine the precise amount of time it was worn each day. Lastly, to facilitate patient compliance with the protocol, the study design does not allow for discontinuation of anticoagulants after the cessation of AF once AF has occurred.

## 5 SUMMARY

The Up to AF trial is an investigator‐initiated multicenter clinical trial designed to investigate whether Apple Watch‐guided personalized anticoagulation therapy, where DOACs are continued or discontinued depending on the presence or absence of AF monitored by the Apple Watch, will reduce the use of DOACs compared to the conventional method of continuous anticoagulation. This will be the first clinical trial to investigate Apple Watch‐guided personalized anticoagulation therapy and provide insights into anticoagulation therapy based on the individual patient's AF status.

## FUNDING INFORMATION

This work was supported by JSPS KAKENHI Grant Numbers JP22K20857 and donations from supporters of the “Research on the best anticoagulant medication for individual patients with atrial fibrillation” project (https://readyfor.jp/projects/handai‐af2023) on the crowdfunding site READYFOR.

## CONFLICT OF INTEREST STATEMENT

None.

## APPROVAL OF THE RESEARCH PROTOCOL

The study protocol and informed consent forms has been reviewed and approved by a certified review board, the Osaka University Clinical Research Review Committee (CRB5180007).

## INFORMED CONSENT

All patients must provide informed written consent prior to participation in this study.

## REGISTRY AND THE REGISTRATION NO. OF THE STUDY/TRIAL

This study has been registered at Japan Registry of Clinical Trials (jRCTs052230070). (URL: https://jrct.niph.go.jp/en‐latest‐detail/jRCTs052230070).

## ANIMAL STUDIES

N/A.

## Data Availability

Our study data will not be made available to other researchers for purposes of reproducing the results due to institutional review board restrictions.
